# Fuelling in front of the barrier—are there age based behavioral differences in Garden Warblers *Sylvia borin*?

**DOI:** 10.7717/peerj.319

**Published:** 2014-03-25

**Authors:** Christos Barboutis, Ian Henshaw, Cecilia Kullberg, Stamatina Nikolopoulou, Thord Fransson

**Affiliations:** 1 Natural History Museum of Crete, University of Crete, Iraklion, Greece; 2 Department of Zoology, Stockholm University, Stockholm, Sweden; 3 Institute of Marine Biology and Genetics, Hellenic Centre for Marine Research, Iraklion, Crete, Greece; 4 Department of Environmental Research and Monitoring, Swedish Museum of Natural History, Stockholm, Sweden

**Keywords:** Bird migration, Sahara crossing, Eastern Mediterranean, Stopover, Crete, Radio tracking

## Abstract

Garden Warblers *Sylvia borin* were studied during autumn stopover in Crete before crossing the barrier of the Mediterranean Sea and the Sahara Desert. Birds followed with transmitters show extensive stopover periods, which were longer in first-year birds, 16 days, compared with adult birds, 14 days. The distribution of body masses from birds trapped in fig trees were used to estimate the departure body mass and the results found indicate that both age categories on average depart with a fuel load close to 100% of lean body mass. The movement of transmitter birds shows differences between first-year and adult birds. Adult birds move further away from the release site and many also left the study area. Several were found settled outside the study area, up to 17 km away, indicating that they regularly make longer stopover movements. It is suggested that this might be a result of that they return to a place where they stayed during an earlier migration. It was shown that stopover site fidelity exists and nine garden warblers were recaptured in the area during a following autumn. The results found highlights the importance of stopover areas close to the Sahara Desert.

## Introduction

About 2 billion songbirds breeding in the Western Palearctic cross the Saharan Desert every autumn to reach their wintering grounds (Hahn, Bauer & Liechti, 2009). The area of Sahara has not always acted as an ecological barrier for migratory birds, and had expanded to a considerable size already a few million years ago (Bruderer & Salewski, 2008). Gradual variations in the extent of the desert has occurred over time, most recently from a humid period to a period of desertification in North Africa seem to have started about 6000 years ago (Holmes, 2008; Kröpelin et al., 2008). These conditions have probably resulted in fluctuations in the difficulty for birds to cross the barrier. Some long distance migrants that choose not to circumvent the Mediterranean Sea have nowadays to cross an ecological barrier that can reach up to 2200 km (Barboutis et al., 2011). Most of the long distance migrants are not adapted to refuel in oases or in the surrounding vegetation (Jenni-Eiermann et al., 2011) and thus extensive fuel loads are stored in advance as can been seen from the very high body masses of birds close to the barrier (e.g., Finlayson, 1981; Fransson et al., 2006; Fransson et al., 2008).

Migrants are expected to optimally modulate their travel costs in terms of time, energy and safety (Alerstam & Lindström, 1990) while crossing diverse geographic sectors, resulting in variation in body mass between different sectors along the migration route (Yohannes et al., 2009). Several species have been shown to be non-randomly distributed between areas close to the Sahara Desert during autumn, indicating that species specific areas are used during preparation for the crossing (Fransson, Jakobsson & Kullberg, 2005). In accordance with this, several species have been shown to exhibit stopover site fidelity close to the barrier (e.g., Moreau, 1972; Cantos & Tellería, 1994; Merom, Yom-Tov & McClery, 2000). Information about spatio-temporal variation in stopover duration and body mass accumulation is crucial in order to understand the organization of the migratory journey of birds (Alerstam & Lindström, 1990). However, detailed information of stopover behavior in songbirds is rare and this is especially evident when it comes to preparation close of the Sahara Desert.

One of the most numerous long-distance migrating passerines in the Palearctic to perform this barrier crossing is the Garden Warbler *Sylvia borin* (Hahn, Bauer & Liechti, 2009). Garden Warblers seem to increase their average body mass during the migration from their breeding grounds to the edge of the barrier in autumn, and this increase was found to be larger in eastern birds (Bairlein, 1991; Schaub & Jenni, 2000). Most of the fuel needed for the barrier crossing, however, is accumulated at their last stopover before the passage (Fransson et al., 2008). The Garden Warbler is known for its seasonal frugivory (Bairlein, 2002) where figs *Ficus carica* provide an important food source when fuelling in autumn in the Mediterranean region (Thomas, 1979; Fransson et al., 2008). Garden Warblers show an age-related difference in timing of autumn migration, where adult birds depart ahead of first-year birds (Fransson, 1995; Barboutis, 2010; Iwajomo, Hedenström & Ottosson, 2012).

In this study we investigate if there are age-related differences in stopover behavior between first-year and adult Garden Warblers in Crete when preparing for the crossing of the Mediterranean Sea and the Sahara Desert in autumn.

## Methods

### The study site

**Figure 1:**
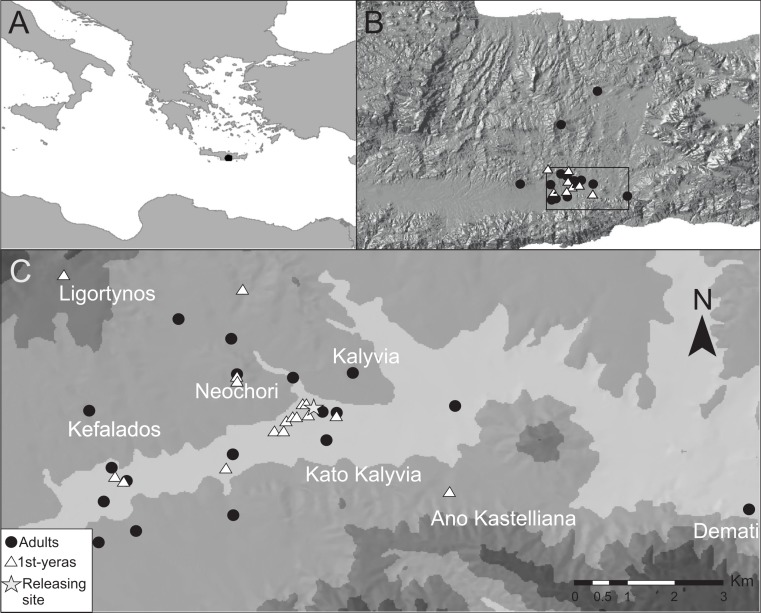
The study site. (A) Study site in relation to the North African coast and the Balkan Peninsula. (B) The study site (black rectangle) in relation to central Crete shown with an altitude relief. (C) A contour map of the study site where light grey areas represent the lowest altitudes. Triangles and circles represent positions during the last day before departure of radio-tagged birds that were followed for a full stopover.

Garden Warblers were studied in south central Crete (Figs. 1A and 1B) during periods of autumn migration between 2004 and 2012 (with exception of 2005). The study area is situated in the neighborhood of the village Kalivia (35°03′N 25°13′E) in the eastern part of the Mesara plains about 20 km from the southern coastline of Crete. The area consists of agricultural landscapes and riverine vegetation along seasonal rivers. Fieldwork was carried out during 2–4 weeks every year and the dates for fieldwork visits varied between years to cover the passage period of the different age-groups in the region according to Barboutis (2010). The earliest start date was 20 August and the latest ended 29 September.

### Trapping and radio-tracking

Garden Warblers were trapped with mist nets either with tape lures started at dawn at one site close to the village of Kalivia or without luring at different fig trees within the valley. Trapped birds were aged according to Svensson (1992) and weighed to the nearest 0.1 g. Maximum wing length (Svensson, 1992) was recorded as a measurement of body size. In total 1113 Garden Warblers were trapped using tape lures, 658 first-year and 445 adult birds, while another 618 were trapped at fig trees without luring, 369 first-year and 249 adult birds.

Light-weight radio transmitters (model BD-2N; Holohil Company Ltd, Canada) were used on 77 Garden Warblers, out of which 29 were first-years (2004 and 2009) and 48 were adult birds (2006–2009). The radio transmitters were attached with Cyanoacrylate glue to trimmed feathers on the back or by leg-loop harness (Rappole & Tipton, 1991) and weighed approximately 0.5 g with a minimum life-span of 21 days. All the birds equipped with radio transmitters were trapped using tape lures at the site close to the village of Kalivia (Fig. 1C). It has been shown that a great proportion of birds trapped with tape lures early in the morning are birds that have performed a migratory flight the night before (Schaub, Schwilch & Jenni, 1999; Fransson et al., 2008) and combined with the fact that we only equipped lean birds with radio transmitters, we believe that the majority of birds studied were newly arrived at the study site. First-year birds were given transmitters between 29 August and 14 September and adults were given transmitters between 21 August and 8 September. Only Garden Warblers with a small amount of visible fat were chosen and the body mass of the transmitter birds varied between 16.2 g and 19.8 g (mean 18.17 g ± 1.01) in first-years and 16.2 g and 19.9 g (mean 17.98 g ± 1.03) in adult birds. Radio-tagged birds were searched for every day in an area of approximately 7 × 10 km (Fig. 1C) using hand held three and four element Yagi antennas or by an antenna attached to the roof of a car. The area outside the study area was sometimes searched for birds that disappeared, but this was for practical reasons not possible to perform regularly. Transmitters were normally detected up to a distance of about 3–4 km. Birds carried the transmitters until they disappeared, but four transmitters fell off (three that were glued and one with leg-harness) and one other had its antenna broken and these were not included in the analysis. Trapping and attachment or radio transmitters were carried out under a licence issued from the Greek Ministry Agriculture and the Hellenic Bird Ringing Centre.

### Departure body mass

When estimating the departure body mass, we have used the median stopover period of birds with transmitters that we have classified to have stayed in the study area until they departed for sub-Saharan Africa (see results), and body mass of birds trapped at fig trees. The method used assumes that birds in fig trees are equally likely to be captured during any given day, and birds trapped at fig trees represent those that have established in the study area (Fransson et al., 2008) and that their body mass is increasing during their stopover. If the mean stopover duration at our site is *χ*, then the mean body mass of the (1/*χ*∗100)% of the heaviest birds represent the departure morning body mass (see Alerstam & Lindström, 1990 for the logic behind this). When calculating the heaviest fraction of birds at fig trees we have included also body mass values from recaptures, which means that a small number of birds are involved with body masses from different days.

## Results

Mean body mass of birds trapped using tape lures on southern Crete was lower for first-year birds compared to adult birds (first-year birds: 19.73 ± 2.38 g, 14.2–29.0, *n* = 636; adult birds: 20.45 ± 2.61 g, 16.2–29.5, *n* = 444; Mann–Whitney: *Z* = −4.277, *p* < 0.001). First-year birds had shorter wings than adult birds (mean wing length of first year birds: 79.9 ± 1.6 mm, *n* = 640 and adult birds: 81.1 ± 1.7 mm, *n* = 445; Mann–Whitney: *Z* = −11.24, *p* < 0.001). Adult birds trapped by means of luring show an increasing body mass against date (Spearman correlation; *R_S_* = 0.15, *p* = 0.001, *n* = 450) while no such correlation was found in first year birds (*R_S_* = 0.03, *p* = 0.437, *n* = 658).

Mean body mass of first-year birds trapped for the first time at fig trees was 21.81 ± 3.56 g (14.6–34.0 g, *n* = 470) and was lower compared with adult birds 23.47 ± 3.26 g (16.1–31.6 g, *n* = 296; Mann–Whitney: *Z* = −6.519, *p* < 0.001).

**Figure 2:**
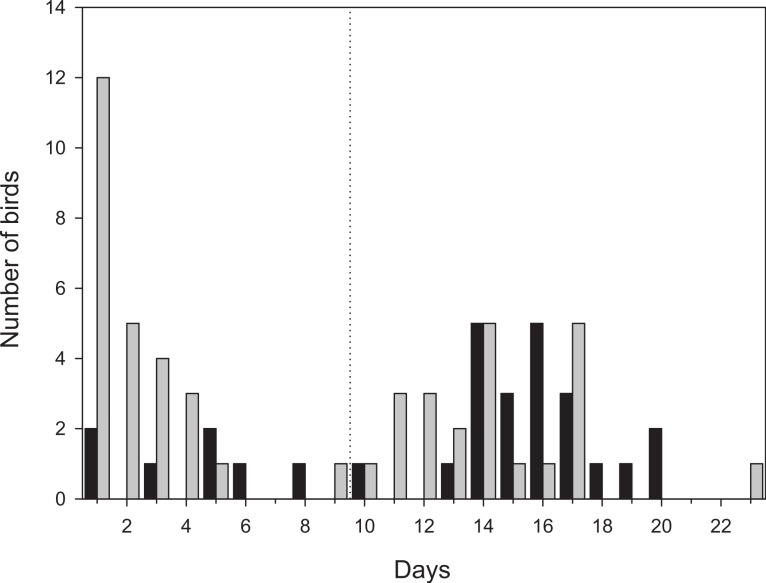
Number of days radio-tagged birds stayed within the study area. Birds that stayed more than 9 days were classified as staying in the area for a full stopover (to the right of the dashed line). Black bars denotes first-year birds (*n* = 29) and grey bars adult birds (*n* = 48). One adult bird that moved outside the study area, but followed until it left after 12 days is included as well.

**Figure 3:**
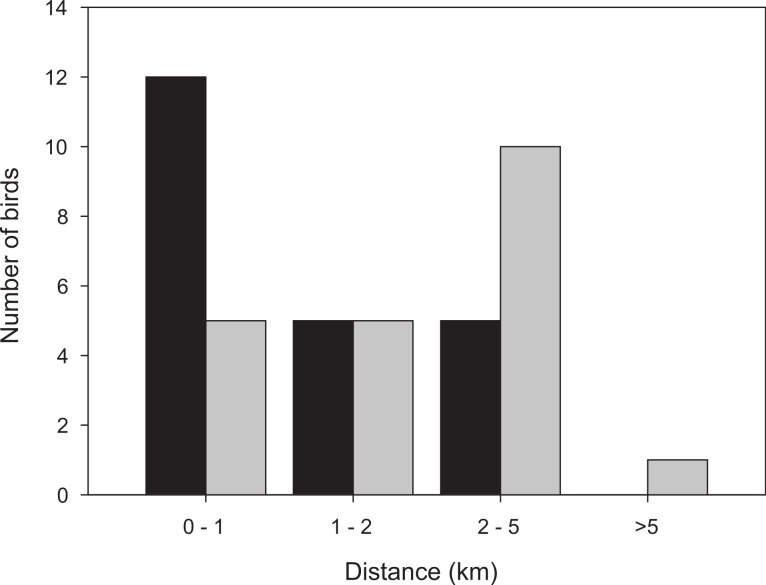
Distribution of distances from the trapping site to the position where the bird spent the last day before take-off, for birds followed for a full stopover within the study area. Black bars: first-year birds, *n* = 22; grey bars: adult birds, *n* = 21.

More than half of the adult birds (54%) disappeared from the study area during the first nine days while the same figure for first-year birds was much less, 24% (Mann–Whitney: *Z* = 3.509, *p* < 0.001; Fig. 2) and only a few individuals disappeared in the interval 7–9 d. Due to the bi-modal distribution of number of days transmitter birds were present in the area (Fig. 2) and flight range estimates indicating that garden warblers have to make a considerable stopover and fuelling to be able to cross the desert (Fransson et al., 2008; Barboutis et al., 2011), we have assumed that those that stayed longer than 9 d made a complete stopover in the study area. The median stopover duration for birds that stayed longer than 9 d was longer for first-years (16 d, *n* = 22) than adults (14 d ± 0.6, *n* = 22; Mann–Whitney: *Z* = −1.993, *p* = 0.046). The median distance from the trapping site to the place where birds spent their last day (Fig. 1C) was shorter for first-years (0.8 km) compared to adults (2.1 km; Mann–Whitney: *Z* = −2.47, *p* = 0.018, Fig. 3). Out of the birds that disappeared from the study area three adult birds were eventually found at 8.5, 10.0 and 16.9 km from the trapping site (Fig. 1B). All of them were recorded in the study area during the day of trapping only and were not found the following morning. They were relocated after three, six and eight days respectively and remained at those places until at least 10–12 days after the initial capture. One of them was followed in detail and left after 12 days and is included in the above calculations of stopover duration.

The morning body mass of the departure day in birds staying longer than 9 d is calculated as the average body mass out of the 1/16 and the 1/14 heaviest fractions of first-year and adult birds trapped at fig trees, respectively. In both cases the estimated average morning body mass during the last day of stopover was 29.5 g (range in first-year birds: 28.1–34.0 g and in adult birds: 28.6–31.6 g). To get body mass conditions in evening, prior to departure, we add one gram giving the estimated evening departure body mass of both first-year and adult Garden Warblers is 30.5 g.

From trapping at fig trees, it is clear that stopover site fidelity between years exists in garden warblers at this stage and nine birds were recaptured during a later autumn in the same area, in several cases in the same fig tree as they were initially ringed. Only two of those were ringed as first-year birds and one of them, ringed in 2009, was recaptured in both 2010 and 2011. Transmitters were attached to four of those during the year of ringing and two were followed in the area for more than ten days.

## Discussion

The results found in this study show that both first-year and adult Garden Warblers make an extensive stopover in autumn before crossing the Mediterranean Sea and the Sahara Desert. The median stopover periods of about two weeks are approximately twice as long as found in Garden Warblers by using capture–recapture methods further north and west in Europe (Schaub & Jenni, 2001). It has, however, been shown when comparing telemetry and mark-recapture methods for estimating stopover duration that the telemetry method could result in much longer stopover durations (Bächler & Schaub, 2007). Adult birds had an estimated stopover period significantly shorter than the one found in first-year birds and such age-related differences have also been found in other species during autumn migration (*c.f.* Ellegren, 1991; Rguibi-Idrissi, Julliard & Bairlein, 2003). Both first-year and adult birds seem to make local movements during the first period of time after arrival, in adult birds this regularly involved leaving the study area while first-year birds, as shown by Fransson et al. (2008), normally stayed within the study area. This means that some birds included in the study might have arrived from other sites on Crete and thus had a longer stopover duration than estimated. Birds chosen for transmitters were however lean, indicating that they were trapped early during their stop over.

Both first-year and adult birds made a considerable fuelling gain during stopover and the estimated departure body mass is close to 100% fuel load compared with the body mass without fat (Ellegren & Fransson, 1992). The method used assumes that birds are equally likely to be captured during any day of stopover when settled at fig trees and that the heaviest birds are found on the departure day (Alerstam & Lindström, 1990). However, Bibby et al. (1976) found that the probability of trapping Sedge Warblers *Acrocephalus schoenobaenus* decreased with increasing body mass. We do not know if this is true also for Garden Warblers, but since they practically only feed on figs (based on analysis of faecal samples; Barboutis C, Palaiothodorou E, Fransson T. 2012, unpublished data) we believe the probability of trapping at fig trees should not change with body mass. Additionally the method used assumes that birds in fig trees are equally likely to be captured during any given day but this assumption is violated. As a portion of birds leave the study area, as shown by telemetry, the estimated departure body mass is likely unaffected as the assumption stands true for birds staying over for more than 9 days which are the birds that show high body mass. Interestingly, the calculated departure body mass found in this study (30.5 g) is very close to the one found using an average calculated fuel deposition rate from re-trapped birds in combination with stopover length on partly the same dataset of first-year Garden Warblers in Crete (30.3 g; Fransson et al., 2008).

Birds trapped with tape lures show that adult Garden Warblers were significantly heavier than first-year birds and if a majority of them are newly arrived (Schaub, Schwilch & Jenni, 1999), adult birds seem to arrive to Crete with larger energy stores than first-year birds. A pattern with heavier adults at sites close to ecological barriers during autumn migration has been described several times both in the Eurasian-African and the American bird migration system (Veiga, 1986; Spina & Bezzi, 1990; Woodrey & Moore, 1997; Yosef & Chernetsov, 2004). This indicates that, at least in some species, age-related migration strategies exist in front of large ecological barriers. The difference found in body mass between adult and first-year birds trapped for the first time at fig trees might also be a result of adult birds arriving to Crete with larger energy stores.

The period of stopover is shorter in adult birds but no difference seems to exist in the departure body mass. Attaining a fuel load that ensures crossing from Crete to the southern edge of the Sahara is most certainly critical and might explain the pattern found in departure body mass. Since carrying fuel loads of the magnitude found in our study entails costs (Alerstam & Lindström, 1990; Kullberg, Fransson & Jakobsson, 1996), we cannot expect birds to have much of a security margin at this point, unlike that which has been suggested for first-year Magnolia Warblers *Dendroica magnolia*, crossing the much shorter ecological barrier of the Gulf of Mexico (Woodrey & Moore, 1997). The distance to pass the Saharan Desert includes stretches of about 2200 km with few or no opportunities for a Garden Warbler to refuel. Barboutis et al. (2011) simulated the desert crossing and found that only one out of 14 transmitter birds with estimated departure fuel loads was unable to reach the southern edge of the desert due to low energy reserves. The bird that did not manage the crossing had an estimated departure body mass of 27.3 g, which is clearly below the range of body masses estimated for birds during their last day of stopover in this study.

Movements of radio-tracked birds during the stopover show differences in stopover behavior between first-year and adult Garden Warblers and a larger proportion of adult birds disappeared from the study area during the first 9 d. Among those that stayed longer and that we believed made a complete stopover in the study area, adult birds spent the last day before departure further away from the initial trapping site than first-year birds. Birds that left the study area shortly after ringing did not carry sufficient fuel loads to cross the Mediterranean Sea and the Sahara, thus it is more likely that they made stopover movements to places outside our study area. The fact that three adult birds were found away from the study area at places where they stayed for more than ten days supports this. In several cases this movement was clearly done by nocturnal flights. This is in line with recent findings that birds during stopover often make nocturnal flights that include several km (Mills et al., 2011; Taylor et al., 2011).

Why are adult birds leaving the study area much more often than first-year birds? The fact that we have recaptured nine Garden Warblers in the same area as they were ringed during a preceding year clearly indicates that some of them are faithful to stopover sites. It might be that some birds are not able to locate the exact stopover site used the previous year but land at another place on Crete. If the new place is not satisfactory (fuel deposition rate, competition etc.) based on expectation from previous years, they may later on relocate to the place where they stayed the previous year. This place might be some distance away, as shown by one adult bird found 17 km north of the release site. First-year birds on their first migration have no prior experience of suitable stopover sites and they also moved shorter distances than adults to find a site where they stayed. Even if many adults left the area after one day, there are still some that leave after a few days. Why this pattern exists we cannot explain at this stage. Stopover site fidelity seems not to be very common in songbirds, but it is interesting that it has been described several times close to the Sahara Desert crossing and the crossing of the Mexican Golf (Moreau, 1972; Cantos & Tellería, 1994; Merom, Yom-Tov & McClery, 2000; Catry et al., 2004; Jubete et al., 2006; Somershoe, Cohrs & Cohrs, 2009; Vogt et al., 2012). Since good stopover sites close to large ecological barriers could be of decisive importance for a successful passage (*c.f.* Fransson et al., 2008) it might be that fidelity at those stopover sites has been advantageous and thus specially favored in front of large ecological barriers.

## Conclusion

In summary our study presents evidence for age-based behavioral differences in Garden Warblers regarding the strategies adopted in preparation for the crossing of a large ecological barrier. Differences found involve stopover duration as well as stopover movements, but in spite of those differences the estimated departure body mass of first-year and adult birds was very similar. Areas close to ecological barriers are critical for many birds to be able to make a successful crossing and as such those areas must be of high conservation interest in the near future. This is especially evident since the ongoing climate change might affect some of those areas very much (Watson, Iwamura & Butt, 2013).

## Additional Information and Declarations

### Field Study Permissions

The following information was supplied relating to ethical approvals (i.e., approving body and any reference numbers): The study was carried out with the permission of the Greek Ministry of Agriculture and the Hellenic Bird Ringing Centre.
